# Anti-inflammatory and Antioxidant Effects of Kaempferia galanga Extract in Acute Bacterial Rhinosinusitis: In Vivo Study on MDA, NF-κB, and CRP

**DOI:** 10.22038/ijorl.2025.90356.4019

**Published:** 2026

**Authors:** Aziza Viquisa Berliana Putri, Paramasari Dirgahayu, Bambangx Purwanto, Soetrisno Soetrisno, Betty Suryawati, Risya Cilmiaty, Hadi Sudrajad

**Affiliations:** 1 *Doctoral Program of Medical Science, Faculty of Medicine, Universitas Sebelas Maret, Surakarta, Surakarta, Indonesia.*; 2 *Parasitology and Micology Department, Faculty of Medicine, Universitas Sebelas Maret, Surakarta, Indonesia.*; 3 *Department of Internal Medicine, Faculty of Medicine, Universitas Sebelas Maret, Surakarta, Indonesia.*; 4 *Department of Obstetrics and Gynecology, Faculty of Medicine, Universitas Sebelas Maret, Surakarta, Indonesia.*; 5 *Department of Microbiology, Faculty of Medicine, Universitas Sebelas Maret, Surakarta, Indonesia.*; 6 *Department of Oral Disease, Faculty of Medicine, Universitas Sebelas Maret, Surakarta, Indonesia.*; 7 *Departement of Otorhinolaryngology, Faculty of Medicine, Universitas Sebelas Maret, Surakarta, Indonesia.*

**Keywords:** Anti-inflammatory agent, Antioxidants, Kaempferia galanga, Oxidative, Rhinosinusitis stress

## Abstract

**Introduction:**

Acute bacterial rhinosinusitis (ABRS) is an inflammation of the paranasal sinuses caused by bacterial infection, primarily involving inflammatory responses and oxidative stress. While antibiotics are the standard treatment, their effectiveness can sometimes be limited. *Kaempferia galanga (K. galanga)*, a medicinal plant known for its anti-inflammatory and antioxidant properties, has shown potential as a therapeutic agent. This study aimed to evaluate the therapeutic efficacy of *K. galanga* extract in a mouse model of ABRS.

**Materials and Methods:**

An in vivo experimental study was conducted using Sprague-Dawley rats with induced ABRS. The animals were randomly divided into three treatment groups that received *K. galanga* extract at doses of 150 mg/kg (KG150), 300 mg/kg (KG300), or 450 mg/kg (KG450) based on body weight. Therapeutic effects were assessed by measuring serum levels of MDA, NF-κB, and CRP.

**Results:**

Eighteen rats with ABRS were included in the study. The KG300 group exhibited significantly lower levels of MDA, NF-κB, and CRP compared to the KG150 and KG450 groups (p < 0.05). These findings suggest that the 300 mg/kg dose of *K. galanga* extract provides optimal therapeutic benefit.

**Conclusion:**

*K. galanga* extract at 300 mg/kg demonstrated the most potent anti-inflammatory and antioxidant effects in a mouse model of ABRS, indicating its potential as an effective alternative or adjunctive therapy.

## Introduction

Acute bacterial rhinosinusitis (ABRS) is a common inflammatory condition affecting the paranasal sinuses, often caused by bacterial infections that exacerbate mucosal inflammation and oxidative stress (1,2). 

The pathophysiology of ABRS involves the overproduction of reactive oxygen species (ROS) and the activation of pro-inflammatory cytokines, leading to mucosal damage and compromised sinus function (3). 

Key biomarkers such as MDA, NF-κB, and CRP are crucial in understanding the severity of oxidative stress and inflammation associated with ABRS (4,5).

The conventional treatment for ABRS mainly involves antibiotics and corticosteroids. However, increasing concerns about antibiotic resistance and the side effects of long-term corticosteroid use highlight the need to explore alternative therapies with anti-inflammatory and antioxidant properties (6). *Kaempferia galanga L.*, commonly known as aromatic ginger or "kencur" in Indonesian, is a medicinal plant traditionally used in Southeast Asian countries to treat various ailments, including respiratory disorders (7).

Phytochemical studies have identified several bioactive compounds in *K. galanga*, especially ethyl p-methoxycinnamate (EPMC), which exhibits significant anti-inflammatory and antioxidant effects. 

EPMC has been shown to inhibit the production of pro-inflammatory mediators such as interleukins and to suppress NF-κB pathway activation, thereby reducing inflammatory responses (8,9). Furthermore, studies have demonstrated the antioxidant potential of *K. galang*a extract, which effectively neutralizes free radicals and reduces lipid peroxidation, as shown by lower MDA levels in various *in vivo* models (10). 

The regulation of oxidative stress by *K. galanga *is crucial, given ROS's role in the development of ABRS (11).

Given these pharmacological properties, this study aims to investigate the therapeutic effects of *K. galanga* extract in a mouse model of ABRS. By measuring levels of MDA, NF-κB, and CRP, we strive to clarify the anti-inflammatory and antioxidant mechanisms of *K. galanga* and provide a scientific basis for its potential as an alternative treatment for ABRS.

## Materials and Methods

### Study Design

This study employed an in vivo experimental design with a post-test-only control group. The research was carried out over 12 months at the Center for Food and Nutrition Studies, Universitas Gadjah Mada, and the Anatomical Pathology Laboratory, Faculty of Medicine, Universitas Sebelas Maret, Surakarta. All animal procedures were performed in accordance with the ethical principles outlined in the Declaration of Helsinki and were approved by the institutional animal ethics committee.

### Induction of ABRS Mouse

A male Sprague-Dawley mouse, 2 months old and weighing 150-200 grams, was used in this study. The animals were obtained from the Center for Food and Nutrition Studies at Universitas Gadjah Mada. Before the experiment, the mouse underwent a 7-day acclimatization period under standardized environmental conditions with free access to food and water. 

To induce ABRS, *S. aureus* inoculum was prepared by incubating bacterial stock cultures in 10 mL of nutrient broth at 37°C for 24 hours. The bacterial suspension was then centrifuged at 3,500 rpm for 15 minutes, and the supernatant was discarded. 

The pellet was resuspended in 10 mL of sterile 0.85% NaCl, resulting in a final bacterial concentration of approximately 3 × 10⁹ CFU/mL. Each mouse received 40 μL of the prepared inoculum intranasally into each nostril using a micropipette. Within 48 hours after inoculation, the onset of rhinosinusitis was confirmed through clinical signs, including nasal discharge, perinasal swelling, labored respiration, behavioral changes, anorexia, pyrexia, and altered vocalization.

### Preparation of Kaempferia galanga Extract

Rhizomes of *Kaempferia galanga* *L.* were collected from Kemuning, Central Java, an upland area at an elevation of 800–1,200 meters above sea level, and harvested at 7 to 10 months of maturity. Extraction was carried out in the phytochemistry laboratory at Dr. Sardjito General Hospital, Tawangmangu. 

The cleaned rhizomes were dried, ground into a fine powder, and 350 g of the powder was macerated in 96% ethanol for 72 hours at room temperature with occasional stirring. The macerate was filtered daily under vacuum, and the filtrate was concentrated using rotary evaporation at temperatures below 50°C to obtain the ethanol extract.

### Experimental Groups

After successfully inducing ABRS, all mice were randomly assigned to three treatment groups (KG150, KG300, and KG450) using a simple randomization method. A computer-generated random-number table was used to ensure equal group sizes (n = 6 per group). Allocation concealment was maintained by labeling cages with numeric codes, and the investigator conducting the biochemical analysis was blinded to group assignments.

Group KG150 received 150 mg/kg body weight, KG300 received 300 mg/kg, and KG450 received 450 mg/kg. 

**Fig 1:**
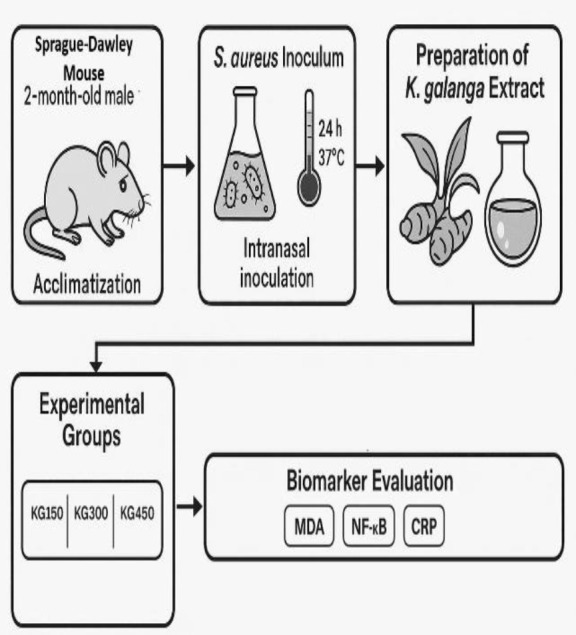
Study Flowchart

The extract was administered once daily for seven consecutive days. On day 8, the mice were euthanized by cervical dislocation for further analysis (Figure 1).

### Biomarker Evaluation

The main outcome parameters included serum levels of MDA, NF-κB, and CRP. 

Blood samples were collected via orbital sinus puncture under light anesthesia. 

The samples were centrifuged to separate the serum, which was then analyzed using enzyme-linked immunosorbent assay (ELISA) kits according to the manufacturer’s protocols.

### Statistical Analysis

All data are presented as mean ± standard deviation (SD). Before performing statistical tests, the data distribution was checked for normality using the Shapiro–Wilk test, and Levene’s test was used to assess the homogeneity of variances. Since the assumptions of normality and equal variances were met, a one-way analysis of variance (ANOVA) was conducted to compare biomarker levels (MDA, NF-κB, and CRP) across the three treatment groups. When the overall ANOVA showed statistical significance, Fisher’s Least Significant Difference (LSD) post hoc test was used to identify pairwise differences between groups. All statistical analyses were performed using IBM SPSS Statistics version 20.0 (IBM Corp., Armonk, NY, USA), with p-values < 0.05 considered statistically significant.

## Results

A total of 18 mice with ABRS were included in the study and evenly divided into three treatment groups. 

The ANOVA test revealed statistically significant differences among the groups for all evaluated biomarkers. Notably, the group receiving 300 mg/kg of K. galanga extract (KG300) consistently showed the lowest levels of MDA, NF-κB, and CRP. These findings are summarized in (Table 1).

**Table 1:** ANOVA test between groups

**Parameters**	**KG 150(n = 6)**	**KG 300(n = 6)**	**KG 450(n = 6)**	**p-value**
MDA	4.91 ± 0.42	3.42 ± 0.04	4.03 ± 0.13	0.001
NF-κB	113.56 ± 7.8	84.33 ± 0.65	95.13 ± 2.16	0.001
CRP	1.1 ± 0.02	0.9 ± 0.01	0.97 ± 0.01	0.001


To better determine the most effective dose, a post-hoc LSD test was conducted. The results confirmed that the KG300 group had significantly lower levels of all inflammatory,oxidative stress, and apoptotic markers compared to the KG150 and KG450 groups. This suggests that administering *K. galanga* extract at 300 mg/kg of body weight is the most effective treatment for reducing ABRS-related pathophysiology. Detailed results of the LSD post-hoc analysis are displayed in Table 2.

**Table 2:** Post-hoc test between groups

**Parameter**	**Group**	**p-value**	**CI 95%**	
			**Lower**	**Upper**
MDA	KG150 vs KG300	0.000	0.98	1.99
	KG150 vs KG450	0.006	0.37	1.39
	KG300 vs KG450	0.027	-1.12	-0.09
NF-κB	KG150 vs KG300	0.000	19.86	38.6
	KG150 vs KG450	0.003	9.05	27.79
	KG300 vs KG450	0.030	-20.18	-1.45
CRP	KG150 vs KG300	0.000	0.17	0.23
	KG150 vs KG450	0.000	0.1	0.17
	KG 300 vs KG450	0.003	-0.1	-0.03

## Discussion

The present study shows that *K. galanga* ethanol extract, especially at a dose of 300 mg/kg body weight, significantly reduces inflammation and oxidative stress in a mouse model of ABRS. These previous results support existing research highlighting the pharmacological potential of *K. galanga* in controlling inflammatory pathways and oxidative stress markers (12,13). 

Inflammation is a key feature of ABRS, characterized by elevated levels of pro-inflammatory cytokines and activation of NF-κB signaling pathways (14,15). In this study, administering *K. galanga *extract resulted in a notable decrease in NF-κB levels, with the most significant effect at 300 mg/kg. This suggests that *K. galanga may exert* anti-inflammatory effects by inhibiting key inflammatory mediators. Previous studies indicated that *K. galanga* contains compounds that reduce the production of pro-inflammatory cytokines. 

A study by Wang showed that *K. galanga* essential oil significantly decreased inflammatory markers in a zebrafish model of oxidative stress, underscoring its potential for treating inflammatory conditions (10). 

Oxidative stress also plays a key role in the development of ABRS, often indicated by increased MDA levels, a marker of lipid peroxidation (16). The current study found a significant reduction in MDA levels after administering *K. galanga* extract, especially at the 300 mg/kg dose, demonstrating its strong antioxidant ability. The antioxidant effects of *K. galanga* are linked to its high flavonoid content, including kaempferol and luteolin, which neutralize free radicals and inhibit oxidative stress pathways (10). These compounds help stabilize cell membranes and prevent oxidative damage in inflamed tissues (13,17,18).

Interestingly, the 450 mg/kg dose of *K. galanga* extract did not further improve biomarker levels and was less effective than the 300 mg/kg dose in this model. This non-linear dose–response may reflect a pharmacodynamic window in which bioactive constituents exert maximal beneficial effects. At higher concentrations, phenolic compounds can display altered redox behavior or cytotoxicity, reducing overall antioxidant and anti-inflammatory effects. Proposed mechanisms in the literature include metal-catalyzed pro-oxidant reactions, redox cycling of phenolics, and metabolic overload of detoxifying systems (19,20). However, further pharmacokinetic and toxicity studies are needed to confirm the dose-related effects observed in this study.

Classical non-steroidal anti-inflammatory drugs (NSAIDs) primarily inhibit cyclooxygenase (COX) enzymes, thereby reducing prostaglandin synthesis and producing anti-inflammatory and pain-relieving effects (21). In contrast, *K. galanga* appears to act mainly by modulating cytokine production and NF-κB signaling, with additional antioxidant activity (10). 

In the study by Samodra & Febrina, an ethanol extract of *K. galanga* was administered orally to male rats before carrageenan-induced paw edema, compared with the positive control, diclofenac sodium. 

The results showed significantly reduced edema at the 5^th^ and 6^th^ hours. They achieved an inhibition rate in the *K. galanga *group comparable to, or in some cases better than, that of the diclofenac sodium group (22). Meanwhile, another study by Jagadish et al. tested various *K. galanga* extracts in both acute and chronic rat inflammation models, finding that *K. galanga* significantly suppressed neutrophil infiltration and thus slowed both acute and chronic inflammation. Although the authors used a standard NSAID, diclofenac sodium, as a reference, the effect of the *K. galanga* extract was strong but did not fully match that of the NSAID in all models (23).

## Conclusion

In summary, this study concludes that *K. galanga* extract at a dose of 300 mg/kg is the most effective treatment due to its anti-inflammatory and antioxidant effects in mice with ABRS. While the study offers valuable insights into the therapeutic potential of *K. galanga* extract in ABRS, we acknowledge several limitations. 

First, the sample sizes in each group were relatively small, which limits the ability to detect more subtle effects. Second, the study relied on systemic serum biomarkers, without histopathological scoring of the sinus mucosa or measurement of local cytokines. Third, we did not use a standard treatment, such as an antibiotic or NSAID, as a positive control, nor did we perform pharmacokinetic or formal toxicity tests to establish safety margins.

Future work should involve histopathological evaluation and local tissue cytokine profiling to confirm effects at the mucosal level. It should also conduct full dose-range and pharmacokinetic/ toxicity studies to determine the therapeutic window and clarify the effects of high doses. Comparing *K. galanga* directly with standard anti-inflammatory agents in head-to-head trials, isolating and testing major active constituents to identify which components drive the observed effects, and assessing chronic and bacterial-clearance outcomes are also necessary. Additionally, exploring combination strategies with antibiotics will help evaluate clinical translatability.

## References

[1] Huriyati E, Darwin E, Yanwirasti Y, Wahid I (2019). Association of inflammation mediator in mucosal and tissue of chronic rhinosinusitis with recurrent nasal polyp. Open Access Maced J Med Sci..

[2] Rapiejko P, Talik P, Jurkiewicz D (2021). New treatment options for acute rhinosinusitis according to EPOS 2020. Otolaryngol Pol..

[3] Tai J, Shin JM, Park J, Han M, Kim TH (2023). Oxidative Stress and antioxidants in chronic rhinosinusitis with nasal polyps. Antioxidants..

[4] Wardani ATW, Purwanto B, Indarto D, Wasita B, Risya Cilmiaty AR (2022). Effect of moringa leaf ethanol extract on reduced levels of MDA, TNF-α and description of inflammatory cells in rat sinus mucosa model of acute rhinosinusitis. Bangladesh J Med Sci..

[5] Cheng N, Wang Y, Gu Z (2023). Understanding the role of NLRP3-mediated pyroptosis in allergic rhinitis: A review. Biomed Pharmacother..

[6] Elshamy AI, Mohamed TA, Essa AF, Abd-El Gawad AM, Alqahtani AS, Shahat AA (2019). Recent advances in kaempferia phytochemistry and biological activity: A comprehensive review. Nutrients..

[7] Wang SY, Zhao H, Xu HT, Han XD, Wu YS, Xu FF (2021). Kaempferia galanga L : Progresses in phytochemistry, pharmacology, toxicology and ethnomedicinal uses. Front Pharmacol..

[8] Umar MI, Asmawi MZ, Sadikun A, Majid AMSA, Al-Suede FSR, Hassan LEA (2014). Ethyl-p-methoxycinnamate isolated from kaempferia galanga inhibits inflammation by suppressing interleukin-1, tumor necrosis factor-α, and angiogenesis by blocking endothelial functions. Clinics..

[9] Dwita LP, Hikmawanti NPE, Yeni Supandi (2021). Extract, fractions, and ethyl-p-methoxycinnamate isolate from Kaempferia galanga Elicit anti-inflammatory activity by limiting leukotriene B4 (LTB4) production. J Tradit Complement Med..

[10] Wang SY, Cai L, Yang N, Xu FF, Wu YS, Liu B (2023). Chemical composition of the Kaempferia galanga L essential oil and its in vitro and in vivo antioxidant activities. Front Nutr..

[11] Patel ZM, Hwang PH, Durand ML, Deschler DG (2018). Acute bacterial rhinosinusitis. Infections of the ears, nose, throat, and sinuses.

[12] Nurmala S, Elfrieda NSAL, Zaddana C, Ali AA (2024). Anti-inflammation effectiveness of ginger (Kaemferia galanga l ) and shallots (Allium ascalonicum l ) extract combination on sprague-dawley male rat. FJIF.

[13] Wang S, Yang J, Cai L, Li H, Han X, Liu B (2025). Antioxidant effect of ethyl acetate fraction from Kaempferia galanga : Integrated Phytochemical profiling, network analysis, and experimental validation. Antioxidants..

[14] Piski Z, Gerlinger I, Nepp N, Farkas K, Weber R (2020). TNF-alpha inhibitors and rhinosinusitis-A systematic review and meta-analysis. Am J Rhinol Allergy..

[15] Tiboc Schnell CN, FILIP GA, Decea N, Moldovan R, Opris R, Man SC (2021). The impact of Sambucus nigra L extract on inflammation, oxidative stress and tissue remodeling in a rat model of lipopolysaccharide-induced subacute rhinosinusitis. Inflammopharmacology..

[16] Hendradewi S, Purwanto B, Dirgahayu P, Wasita B, Pamungkas EP (2020). The effect of Escherichia Coli induction on superoxide dismutase (SOD) and malondialdehyde (MDA) levels in acute rhinosinusitis white rats models. Bali Med J..

[17] Alrumaihi F, Almatroodi SA, Alharbi HOA, Alwanian WM, Alharbi FA, Almatroudi A (2024). Pharmacological potential of kaempferol, a flavonoid in the management of pathogenesis via modulation of inflammation and other biological activities. Molecules..

[18] Herrera TES, Tello IPS, Mustafa MA, Jamil NY, Alaraj M, Atiyah Altameem KK (2025). Kaempferol: Unveiling its anti-inflammatory properties for therapeutic innovation. Cytokine..

[19] Sotler R, Poljšak B, Dahmane R, Jukic T, Jukic DP, Rotim C (2019). Prooxidant activities of antioxidants and their impact on health. Acta Clin Croat..

[20] Nowak M, Tryniszewski W, Sarniak A, Wlodarczyk A, Nowak PJ, Nowak D (2022). Concentration dependence of anti- and pro-oxidant activity of polyphenols as evaluated with a light-emitting Fe2+-Egta-H2O2 system. Molecules..

[21] Stiller CO, Hjemdahl P (2022). Lessons from 20 years with COX-2 inhibitors: Importance of dose-response considerations and fair play in comparative trials. J Intern Med..

[22] Samodra G, Febrina D ( 2020 ). The comparison between anti-inflammatory effects of ethanol extract from Kaempferia galanga L. and diclofenac sodium induced by carrageenan. 1st International Conference on Community Health (ICCH 2019).

[23] Jagadish PC, Latha KP, Mudgal J, Nampurath GK (2016). Extraction, characterization and evaluation of Kaempferia galanga L (Zingiberaceae) rhizome extracts against acute and chronic inflammation in rats. J Ethnopharmacol..

